# Stents in Renal Artery Bifurcation Stenosis: A Case Report

**DOI:** 10.1155/2011/653143

**Published:** 2011-07-12

**Authors:** Polytimi Leonardou, Paris Pappas

**Affiliations:** Department of Radiology, Laikon General Hospital of Athens, 115 27 Athens, Greece

## Abstract

A 39-year-old patient presented with poorly controlled hypertension, and she was referred to renal angiogram and potential renal angioplasty. Renal angiogram showed a bifurcation lesion of the right renal artery. A guide wire was used to cross the upper branch, while the lower branch was protected by another same-type guide wire through the same introducer. Two thin monorail balloons were used to dilate the two branches; however, despite balloon dilatation, the stenosis of the vessels persisted. The “kissing balloon” technique was then attempted by simultaneously inflating both branches using the same balloons, but more than a 70% residual stenosis persisted in each branch. Two stents were finally placed in a “kissing” way through the main renal artery. The imaging and clinical results were good, without any procedure-related complications. Three years clinical followup was also good, without any reason for further interventional approach.

## 1. Introduction

Severe renal artery lesions, especially ostial ones, have been proved to be effectively managed by percutaneous intervention with balloon expandable stents, resulting in enhancement of the renal blood flow and control of blood pressure [1]. Approach to revascularization is complicated when renal artery bifurcation is involved, since there is a possibility of creating a total side-branch occlusion during balloon expansion [2]. 

 We hereby report a case of stenosis at the renal artery bifurcation treated by employing percutaneous balloon dilatation and stenting of both renal artery branches in a sequential fashion with excellent angiographic imaging and clinical results. Technical aspects for this procedure in the management of renal artery bifurcation stenosis are also discussed.

## 2. Case Presentation

### 2.1. Medical History

 A 39-year-old female presenting with headache was noted to have hypertension with a blood pressure of 170/100 mm Hg. The patient had normal serum cholesterol and lipid profile, normal CRP rates, and was a nonsmoker. There was no history of diabetes and no family history of atherosclerosis. Serum creatinine (112 *μ*mol/L) was within the normal range (82–126 *μ*mol/L). Despite initial treatment with two antihypertensive agents, her blood pressure remained poorly controlled 6 months later with a systolic blood pressure of 150 to 170 mm Hg and a diastolic blood pressure of 100 to 115 mm Hg. A captopril Tc-labeled diethylenetriaminepentaacetic acid (DTPA) scan was positive for right renal artery stenosis, whereas the Doppler Ultrasound examination revealed a normal length (9.8 cm) right kidney with a resistive index (RI) of 0.52 at the segmental renal arteries. In view of the patient's relatively young age and poorly controlled hypertension, she was referred for renal angiogram and potential renal angioplasty.

### 2.2. Technique of the Procedure

**Figure 1:**
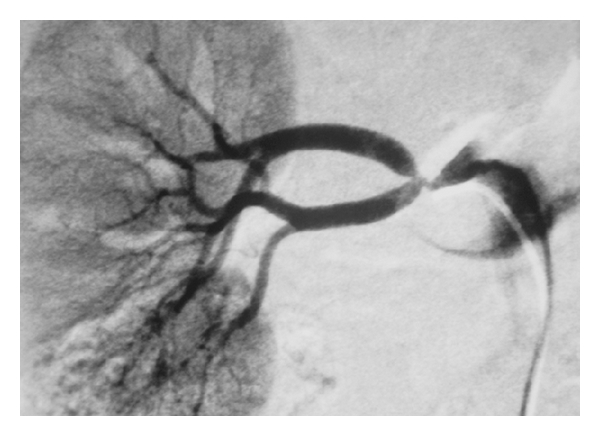
Bifurcation stenosis of the right renal artery.

**Figure 2:**
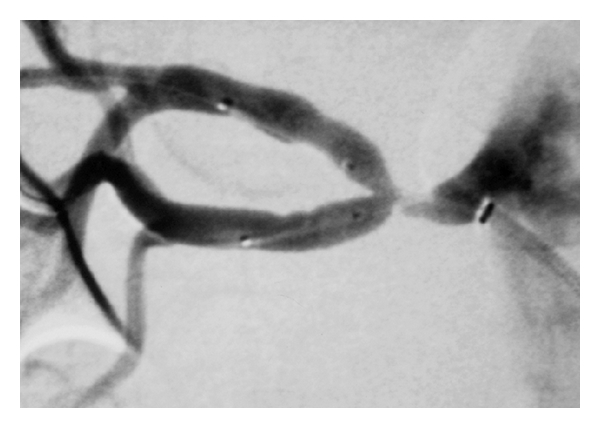
Postdilatation image with two monorail balloons through the same introducer (deflated balloons can be seen into the renal artery branches).

Arterial access was succeeded by a vascular access catheter needle, an angled 0.035 inch–180 cm hydrophilic guide wire, and a 5 F–10 cm introducer. Renal angiogram performed with a 5 F Cobra catheter showed a bifurcation lesion of the right renal artery (Figure 1). The left renal artery had a normal appearance. Percutaneous transluminal angioplasty of the right renal artery was decided. An intravenous bolus dose of 5,000 units of heparin was given. The right renal artery was engaged with a 7 F–65 cm long vascular sheath. A 0.014 inch–180 cm guide wire was used to cross the upper branch, while the lower branch was protected with another same-type 0.014 inch guide wire, both through the same introducer. Two 3 mm–15 mm monorail balloons (Mercury, Abbott) were used to dilate the two branches, first at the nominal pressure of 8 atmospheres for one minute and again for another minute at 12 atmospheres (maximal pressure 16 atmospheres). Despite balloon dilatation, the stenosis of the vessels persisted. The “kissing balloon” technique was then attempted by simultaneously inflating both branches using the same balloons at the same pressures. Despite this, there was more than a 70% residual stenosis in each of the branches (Figure 2). Stents placement was decided for this particular case in a “kissing” way through the main renal artery with two 3 mm–15 mm monorail balloon expandable stents (Vision, Abbott) inflated at the nominal pressure of 9 atmospheres (maximal pressure 16 atmospheres). 

**Figure 3:**
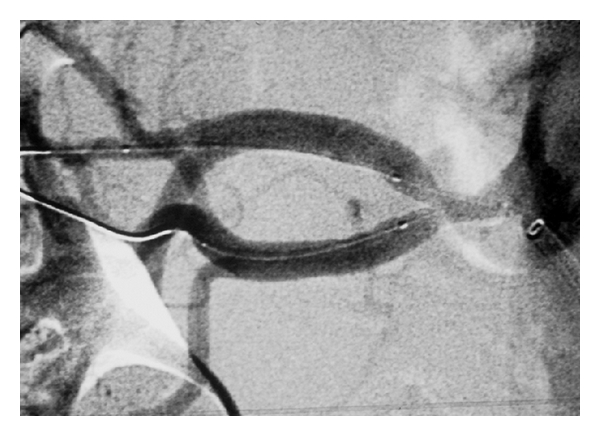
After two-balloon dilated stent placement image, with a good angiographic imaging result.

 Good angiographic results were finally achieved (Figure 3). No further poststenting balloon dilatation was needed. Heparin was discontinued, and the patient was transferred to an intermediate care unit overnight. By the next day, the patient's blood pressure was 120/70 mm Hg without antihypertensive medications, and she was discharged home on aspirin 300 mg per day and ticlopidine 250 mg bid for 4 weeks. 

 The patient remained well with a blood pressure of 130/80 mm Hg at 1-year followup. Doppler ultrasound revealed an RI of about 0.80 in a normal length kidney. Repeat captopril DTPA scan showed good perfusion in both kidneys with no differential renal function or delay in transit time. 

 According to the literature, angiographic success is defined as achieving 30% final diameter stenosis for all treated lesions in any given patient [3–5]. Clinical success is defined as normalization of the blood pressure (systolic <150 mm Hg and diastolic <90 mm Hg), while the patient receives the same or fewer medications without a major procedure-related complication. Major complications include stent-related death, myocardial infarction, bleeding requiring transfusion or vascular surgery repair, renal failure requiring dialysis therapy, emergency renal artery bypass surgery, or stent thrombosis within 30 days of the procedure. Angiographic restenosis is defined as 50% diameter stenosis at the site of stent placement.

 In our successful case, the imaging and clinical results were good, without any procedure-related complications. The three years clinical followup with blood pressure measurement and Doppler ultrasound of the renal arteries was also good, with normal renal function and with no reason for any further interventional procedure.

## 3. Discussion

Renal artery stenosis is a common cause of secondary hypertension [2, 4, 6]. Severe stenosis may lead to loss of excretory function of the kidney. Atherosclerotic lesions are primarily ostial or proximal in location. These lesions are often eccentric, elastic, and respond poorly to balloon angioplasty alone with significant residual stenosis and restenosis. Stent placement in this context has been shown to improve immediate and long-term outcome [1, 3, 4]. 

 Percutaneous transluminal balloon angioplasty is an accepted treatment for selected renal artery stenoses caused by fibromuscular dysplasia [6]. Stent placement appears to be a very attractive therapy in patients with lesions difficult to treat with balloon angioplasty as well as after a suboptimal balloon angioplasty result. Results after renal artery stent placement have demonstrated superior hemodynamic and angiographic outcomes when compared with published results with balloon angioplasty alone [6–8].

 The exact aetiology of the renal artery stenosis in our case is unclear. The lesion lacks the “string-of-beads” appearance typical of fibromuscular dysplasia. The poor response to balloon angioplasty despite the kissing-balloon technique is also atypical for fibromuscular dysplasia. On the other hand, the patient's relatively young age, lack of any atherosclerotic risk factors, and the mid-segmental location of the lesion suggest fibromuscular dysplasia to be the cause.

 Bifurcation lesions have always been a taxing problem in coronary angioplasty. A common approach to bifurcation lesions is the double guide wire sequential dilatation technique [9]. With the second guide wire protecting one of the branches, dilatation is performed sequentially in each of the branches. This, however, does not prevent plaque shifting or side-branch narrowing. In the kissing-balloon technique, balloons are inflated simultaneously in a kissing pattern to prevent plaque shifting and side-branch narrowing [10, 11]. This technique also allows optimal dilatation of larger caliber proximal vessel without overdilating smaller vessel distal to the bifurcation. In this case, stenting the bifurcation lesion with the stent following the “kissing stent” technique achieved a good immediate and long-term result. The result also goes along with that referred in the literature, where normal length (9.8 cm) and low RI (0.52) before angioplasty correlate well with a good outcome [12].

## 4. Conclusion

Percutaneous transluminal angioplasty and stenting technique is a reliable and safe method for treating bifurcation stenosis of the renal artery with stable long-term results, presenting with improved clinical and laboratory results.

##  Consent

Written informed consent was obtained from the patient for publication of this case report and the accompanying images. A copy of the written consent is available for review by the Editor-in-Chief of this journal.

##  Conflict of Interests

Both authors declare that they have no conflict of interests.

##  Authors' Contributions

P. Pappas treated treated the patient and interpreted the patient data. P. Leonardou was assisting during the procedure and contributed to the writing and editing of the paper. P. Pappas was the general consultant and advised in the management of the case and writing of the paper. Both authors read and approved the final paper.
